# Stair-climbing wheelchair proven to maintain user’s body stability based on AnyBody musculoskeletal model and finite element analysis

**DOI:** 10.1371/journal.pone.0279478

**Published:** 2023-01-26

**Authors:** Yancong Zhu, Haojie Li, Shaojun Lyu, Xinying Shan, Yih-Kuen Jan, Fengling Ma

**Affiliations:** 1 School of Biological Science and Medical Engineering, Beihang University, Beijing, China; 2 School of P.E and Sports, Beijing Normal University, Beijing, China; 3 Department of Rehabilitation Technical Aids for Old-Age Disability, National Research Center for Rehabilitation Technical Aids, Beijing, China; 4 University of Illinois at Urbana-Champaign Champaign, Champaign, IL, United States of America; Complutense University of Madrid: Universidad Complutense de Madrid, SPAIN

## Abstract

The electric stair-climbing wheelchair is a beneficial mobile assistance device for older adults and disabled persons with poor walking ability, as it reduces the daily walking and climbing burden. In this paper, 11 older adults were tested when using a stair-climbing wheelchair in three environments: flat ground, slopes, and stairs. The kinematic and dynamic parameters of the lower limb joints were simulated by AnyBody 7.2 human model simulation software using Vicon 3D infrared motion capture, a 3D force table, and analyzed by ANSYS 19.2 Workbench. The joint force, joint moment, and muscle strength did not change significantly under the three environments when using the wheelchair. Through finite element analysis of the mechanical properties of the human body, when using the wheelchair, no significant differences in the overall stress distributions of the fifth lumbar spine, hip bone, or femur were found among the three environments, no significant differences in deformation and displacement were found, and the stress distribution was relatively stable. Therefore, the human body is stable enough to use the electric stair-climbing wheelchair in the three test environments, all of which will be commonly encountered in daily life.

## 1. Introduction

The aging of the population is a global trend, and it is becoming a global challenge. In 2019, the 26th World Population Prospects Report of the United Nations noted that the world population is aging continuously. Older adults aged 65 and above accounted for one-tenth of the total population in 2019, and as the fastest-growing age group, are expected to account for one-sixth of the total population in 2050 [1]. According to the seventh census of China conducted in 2020, China’s population has exceeded 1.4 billion people, of which 264 million are over 60 years old, accounting for 18.7% of the total population; this is an increase of 5.44 percentage points compared with 2010. Some studies predict that the trend of population aging will continue in the next 10 years and peak in 2059, when there will be one older adult out of every three people [2]. Unfortunately, aging is accompanied by increased risk for many diseases and disabilities. Owing to a decline in physiological function or the influence of cardiovascular and cerebrovascular diseases, many older adults have difficulties standing and walking and face various challenges in their daily lives. For older adults, stair climbing is one of the most challenging and dangerous activities of daily life [3]. Jensen [4] noted that stair climbing is faced daily and is a critical aspect of community life. Many older adults and people with certain disabilities need to use crutches or wheelchairs for assistance with walking and climbing stairs.

Wheelchairs are an essential mobility device for many older adults and physically disabled people. Electric wheelchairs have been widely used for many years. Today, it is quite common for an electric wheelchair to be a person’s primary mobility device to aid in daily activities, both indoors and outdoors [5]. However, it is still challenging for a standard electric wheelchair to overcome environmental barriers, such as stairs in buildings or civil infrastructure [6], especially for people living in buildings without elevators. Studies have shown that going uphill and climbing stairs are critical obstacles for wheelchair users, and it is difficult for older wheelchair users to overcome steps with a height of more than 20 cm [7]. Most electric wheelchairs require external assistance when traveling uphill and climbing stairs, so older adults may face risks when attempting to travel uphill independently. In addition, elevators cannot be used during disaster evacuations, such as for fires, earthquakes, and other disasters. Therefore, for the safety of wheelchair users, the ability to travel up and down stairs is a vital issue.

To solve these problems, many designers have designed age-appropriate wheelchairs with usefulness and safety as the core principles [8]. For example, Erwin Prassler’s WHEELESLEY wheelchair adopts advanced human–computer interaction and environment perception technology, and it achieves intelligent obstacle avoidance [9]. Yi et al. successively designed intelligent wheelchairs based on surface electromyography (EMG), lip control, and wrist potential control [10–12]. However, these studies mainly focused on four aspects: motion control of the automatic platform, unique assistive technology, environmental awareness and navigation systems, and human–computer interaction technology [13]. Studies on the stability of stair-climbing wheelchairs are limited to the mechanical structural design and dynamic analysis of wheelchairs, and there are few studies on the stability of older wheelchair users, especially their physical stability, during stair climbing.

The aging process involves physiological and musculoskeletal changes, and the muscle strength of the lower limbs decreases with age [14, 15]. Wheelchairs tilt to various angles while climbing up and down slopes and stairs, which may cause body instability and reduce the comfort of the wheelchair user. In addition, when wheelchairs move from a flatter surface to a steeper surface, the impact force caused by the change in the acceleration of the wheelchair may cause instability in the lower limbs. However, studies in these areas are rare. Therefore, based on a human body dynamics finite element analysis, we used the biomechanical simulation software AnyBody and ANSYS Workbench to conduct inverse dynamics calculation and infinite element analysis on the lower limb joint force, joint moment, and muscle strength of older adults using an electric stair-climbing wheelchair on flat ground, slopes, and stairs. The dynamic data on the joint force, joint moment, and muscle strength were analyzed using inverse kinematics (IK), and the data on the ischium and femur were analyzed using finite element stress and strain analyses. A comparative analysis helped us find the different biomechanical mechanisms of the body during the different uses of the electric stair-climbing wheelchair. This study can provide a theoretical basis for verifying the efficacy and evaluating the ride comfort of electric stair-climbing wheelchairs.

## 2. Materials and methods

### 2.1 Ethics statement

All methods in this study were performed in accordance with the Declaration of Helsinki relevant guidelines and regulations. This study was reviewed and approved by the Ethics Committee of the China National Rehabilitation Center (No. S20220206). The participants provided written informed consent to participate in this study. The potential risks and benefits of participation in this study were explained to each participant in advance. All participants provided signed informed consent before participation.

### 2.2 Participants

Eleven individuals (11 females) from the National Research Center for Rehabilitation Technical Aids of China participated in the study. Participants were recruited based on their age and physical condition. They were in good physical condition and had had no injuries to their lower extremities, ankle, knee, or hip joint within the past six months. Their dominant leg was on the right side. After confirming study eligibility, participants provided informed written consent for the study protocol as approved by the Office of Research Ethics at the National Research Center for Rehabilitation Technical Aids of China. The basic information of the participants is shown in Table 1.

**Table 1:** Anthropometric details of participants (mean ± standard deviation).

Number of participants	Sex	Age (y)	Height (cm)	Mass (kg)
11	Female	50.82 ± 7.62	160.36 ± 4.67	63.27 ± 6.04

### 2.3 Instrumentation

**Fig 1:**
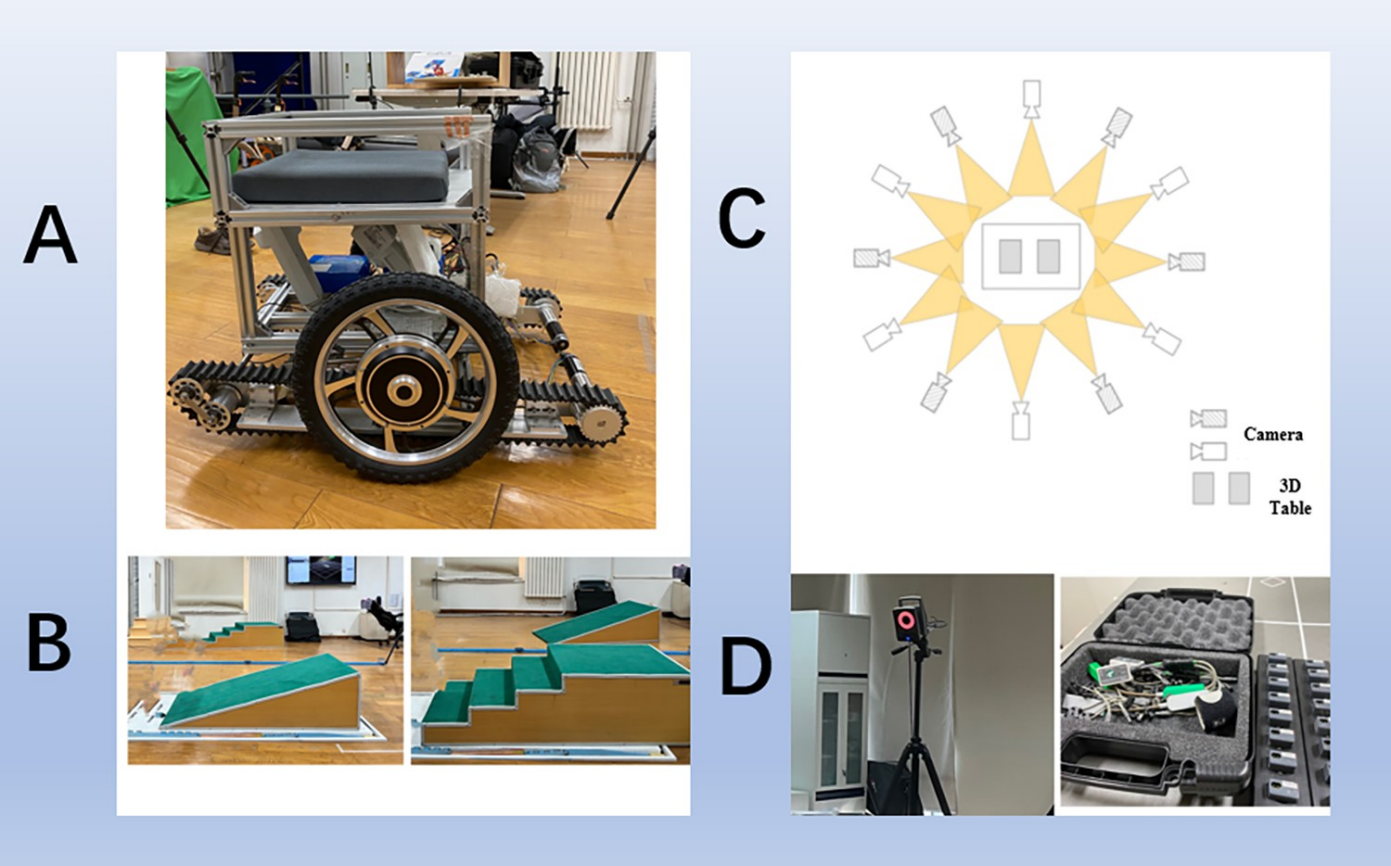
(A) Electric stair-climbing wheelchair used in the test. (B) Simulated slope and stairs. (C) Field test environment diagram. (D) VICON infrared motion-capture equipment and surface EMG equipment.

In this study, the electric stair-climbing wheelchair was a wheel-track composite wheelchair, as shown in Fig 1A. This wheelchair fully combines the advantages of a wheel mechanism and track mechanism and can run like an ordinary electric wheelchair when on flat ground. When encountering stairs or obstacles, the track can be lowered to become a climbing mechanism. This device is the stair-climbing wheelchair. The mechanical system of the wheelchair is mainly divided into a crawler drive module, balance car module, automatic support mechanism of the crawler drive module, auxiliary wheel and its automatic support mechanism, design and layout of the sensor system, and whole-frame structure. The specifications of the stair-climbing wheelchair in this experiment were as follows: dimensions of 876 mm × 750 mm × 650 mm, maximum speed on the ground of 1.5 m/s, maximum speed on stairs of 0.3 m/s, climbing capacity of 33.6°, stair width of ≤300 mm, and stair height of ≤200 mm.

The staircase used in the experiment is shown in Fig 1B, with a width of 260 mm, a height of 170 mm, and a slope of 33.18°, which conforms to the stair slope and step specifications in the Chinese civil building design code [16].

We selected twelve Vicon T20S cameras as data acquisition equipment for kinematic shooting. To ensure the accuracy of data acquisition, six cameras were mounted, and six cameras were scaffolded, as shown in Fig 1C. The specifications of the cameras were as follows: produced by OML, UK; resolution of 2352 × 1728 pixels, 4 million pixels, and acquisition frequency of 100 Hz. Two 3D force-measuring tables (928E, Kistler, Switzerland) were used to collect the surface reaction force data with a sampling frequency of 1000 Hz and a static detection error of less than 0.5%. The experimental equipment are shown in Fig 1D.

### 2.4 Testing protocol

First, we adjusted the VICON camera positions. The mounted and scaffolded cameras formed a semiarc around the center of the test area (Fig 1C). The distance between each camera and the simulated stairs was 2 m. Before the experiment, we debugged the system to check whether the equipment could run normally and ensure that each camera could capture a picture simulating the staircase. Calibration included calibration of the global coordinate system in space and calibration of the position of the force platform.

The study participants wore uniform black elastic short sleeves, shorts, and black socks to reduce the introduction of error by other variables, and 25 reflective marker dots were attached to the participants according to the location of the marker dots on a bone–muscle model. The positions of the marker points included the left and right anterior head, left and right posterior head, sternum, clavicle joint, 10th thoracic vertebra, sternal xiphoid process, left and right anterior superior iliac spine, left and right posterior superior iliac spine, left and right lateral lower one-third of the thigh, left and right external epicondyle of the fibula, right lateral lower one-third of the calf, left lateral lower half of the leg, left and right heel, left and right lateral malleolus, left and right first metatarsal, and left and right fifth metatarsal [17, 18].

**Fig 2:**
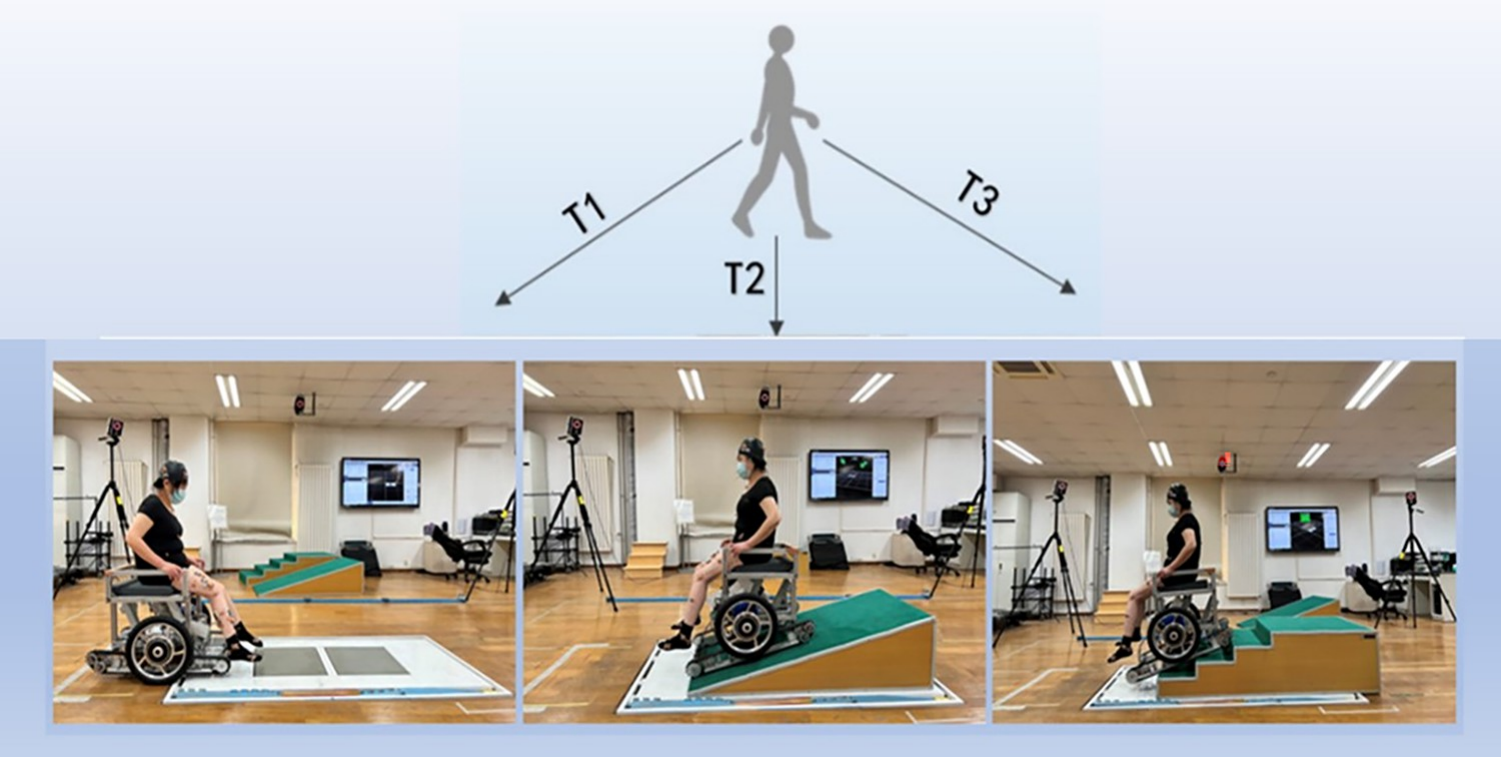
Test scheme. T1: flat ground environment, T2: slope environment, T3: stair environment.

As shown in Fig 2, the participants traversed the ground, slope, and stairs by using the wheelchair, and the Vicon cameras captured their motion.

### 2.5 Data processing and analysis

In this study, data were collected by a BTS 3D infrared-based motion capture system and Kistler 3D force plate. Data calculation was conducted by AnyBody 7.2 musculoskeletal modeling. Infinite element analysis was performed using ANSYS 19.2 Workbench (Cybernet Systems Co., Ltd., Tokyo, Japan).

The biomechanical simulation software AnyBody 7.2 was used for the 3D motion-capture dynamics analysis. The muscle forces were solved by inverse dynamics simulation, which calculates and specifies the mode of muscle activity through which the desired motion is accomplished [19, 20].

#### 2.5.1 Skeletal muscle model of the lower limbs

**Fig 3:**
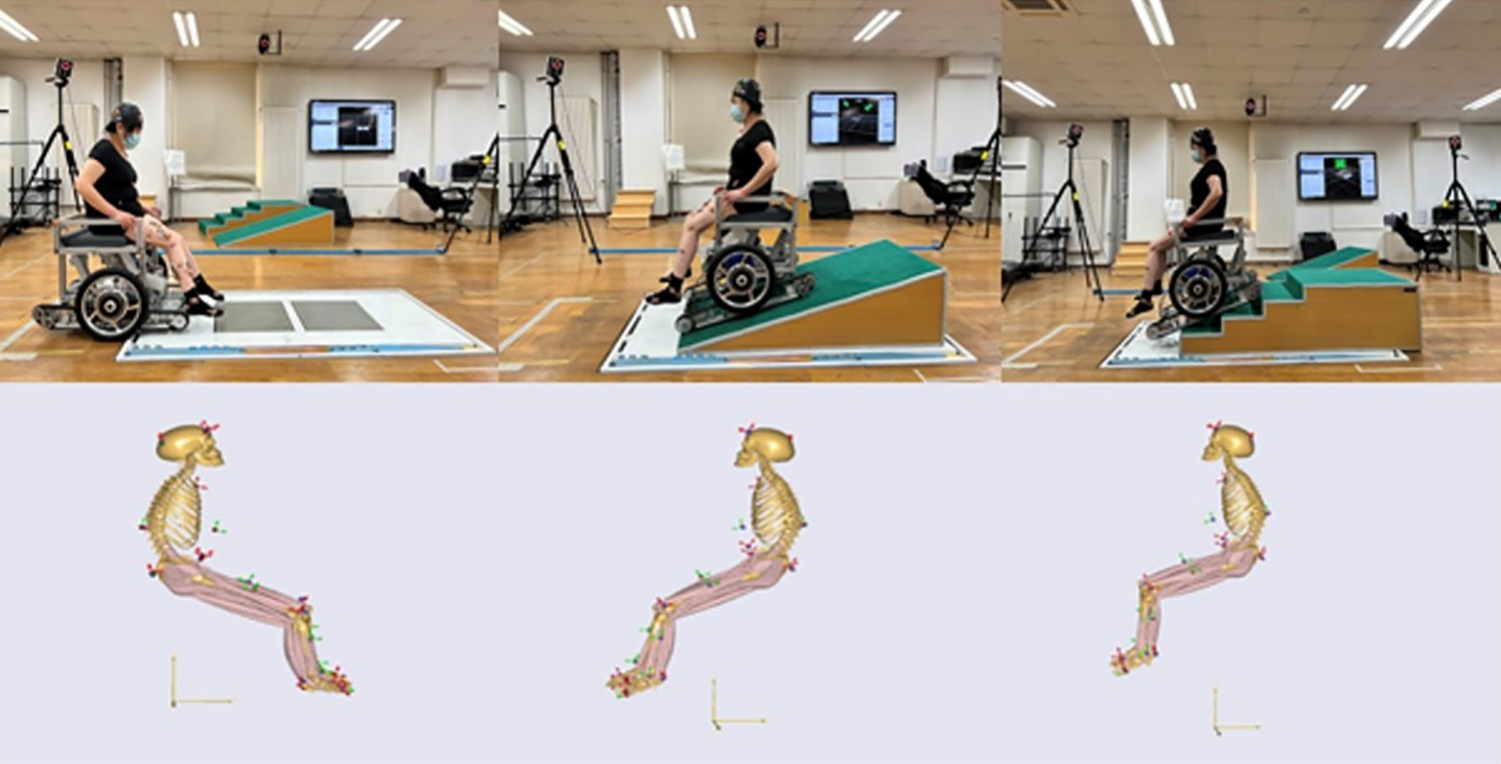
Musculoskeletal models of a subject using the wheelchair in the three test environments.

A new lower limb model was built based on the lower limb model in AnyBody by Vicon Nexus 2.6.1. Data were collected from the lower limb movement model. We used 12 Vicon T20S cameras to model the movement data of reflex markers on the participants and exported the 3D files. Then, we imported the 3D files into AnyBody 7.2 to build skeletal muscle simulation models of the subjects using wheelchairs in the three test environments (Fig 3).

The skeletal muscle model constructed in AnyBody is a standard multibody dynamic model consisting of a rigid body, kinetic pairs, kinematic actuators, and force/moment actuators, such as muscles. The force or moment obtained is solved mainly through multibody dynamics simulation [21].

The muscular system usually contains more muscles than are needed to drive the joints. This problem is usually solved by the central nervous system (CNS), which controls the activation/recruitment of muscles. The AnyBody modeling simulation system provides a standard mechanism for selecting optimal muscle systems to simulate how the CNS works:

$$Minimize\;G(f^{\left(M\right)}),$$ (1)

Cf=d, (2)

$$0\leq f_{i}^{\left(M\right)}\geq N_{i},\;i\in \{1,\ldots ,n^{\left(M\right)}\},$$ (3)

where G is an objective function of the distribution strategy of the assumed CNS to the muscle stress; $f_{i}^{\left(M\right)}$ is the muscle force of the external equilibrium load, and it is the dependent variable; *Cf* = *d* is the dynamic balance equation solved by the human muscle model; *C* is the matrix of the equation system; *d* is the sum of inertia force and external force; and N_*i*_ is the strength of the muscle to be calculated.

The calculation formula of muscle recruitment is as follows:

$$G(f^{\left(M\right)})=\sum_{i=1}^{n^{\left(M\right)}}{\left(\frac{f_{i}^{\left(M\right)}}{N_{i}}\right)}^{p},$$ (4)

where *p* is a polynomial power series (*p* ≥ 1). When *p* is set to different values when the power series is large, the number of muscles balancing the external force will be higher, and the synergy effect will be significant [22]. When the muscle model or human physiological system is in such a balanced state, due to the collective protection mechanism, the maximum relative load on muscles is required to be minimal. At this point, the muscle activity is minimal to avoid muscle strain caused by excessive stress:

$$max\left(\frac{f_{i}^{(M)}}{N_{i}}\right),i=1,2,3,\ldots ,n^{\left(M\right)}.$$ (5)

The calculated dynamic indices were imported into Excel for standardized processing: in the first treatment, joint force and joint moment were divided by body weight (unit: N/BW) [23]; in the second treatment, the muscle strength of the lower limbs was divided by body weight and multiplied by 100% (N/BW) [24].

#### 2.5.2 Bone finite element analysis

We established a finite element model of bones based on CT scans of the lumbar spine, hip, and femur of a healthy middle-aged woman (56 years old, 162 cm, 65 kg). The model included the fifth lumbar vertebra, the left and right hipbones, and the femur of the right leg. We imported the original DICOM image of bone CT data into Mimics 20.0 (Materialise, Leuven, Belgium), extracted the bone, and exported the STL format by dividing the finite bone element into tetrahedrons. The bone and the retrograde mesh were restored by Geomagic Wrap 2013 (Geomagic Company, Research Triangle Park, NC, USA), which was used to fill the holes and repair sharp corners, and the IGES format was exported after the treatment was completed. Finally, the Young’s modulus and Poisson’s ratio of the fifth lumbar vertebra, hip bone, and femur were added to ANSYS 19.2 Workbench to perform constraint fixation on the three bones. Toyohara’s study demonstrates that Young’s modulus is 11,000 MPa and Poisson’s ratio is 0.2 [25]. Moreover, all tissues were defined as uniform isotropic material for simplification and their material properties were taken from the experimental results by Wirtz et al. [26]. Gridding method: we created grid skeletons and joints with tetrahedral elements, each of which was composed of 10 nodes. The average quality of elements was 0.75, indicating that the grid was of good quality. As a comprehensive index for quality, element average quality ranges from 0 to 1, with values of 0 and 1 respectively representing an element with zero volume and a perfect cube.

Load and boundary conditions: We conducted this experiment in a static simulation environment. To correctly simulate the conditions where participants walk and sit in a wheelchair, loads on finite element models were calculated by AnyBody’s inverse dynamics. Loading force refers to joint force at the joint connection, whereas loading facet refers to the articular facet at the joint connection specified by AnyFE2Abq plug-in of AnyBody.

Variables measured: this study focused on the structural displacement and von Mises stress of three bones. The former is composed of a three-dimensional displacement value and direction, and the latter is a scalar value from normal stress and shear stress with no distinction between tension and compression.

The net joint force was loaded to the upper articular surface of the lumbar spine, the lumbosacral articular surface of the hip bone, and the femur trochanter, and then the force and deformation displacement of the bone were analyzed.

### 2.6 Statistics

All analyses were performed with statistical analysis software SPSS (SPSS Version 26.0, SPSS Inc., Chicago, IL, USA). The results are expressed as the mean and standard deviation (mean ± SD). T-test and analysis of variance were used to compare the dynamic data of the three movements in different environments. Differences were statistically significant if P < 0.05.

## 3. Results

### 3.1 Dynamic comparisons of three lower extremity joints in three environments

#### 3.1.1 Stress comparisons on three joints of the lower limbs in three environments

As shown in Table 2, the X, Y, and Z forces of the hip, knee, and ankle joints were measured when using wheelchair. there were no significant changes in the joint load on the hip, knee, or ankle joints in slope and stair environments compared to flat ground environments.

**Table 2:** Mean and standard deviation (mean ± SD) of the maximum joint force of the hip, knee, and ankle in three environments. Units: N/BW.

Joint force	Direction	Flat ground	Slope	p-value	stairs	p-value
Hip joint	X-axis	−0.03 ± 0.02	−0.07 ± 0.01	0.274	−0.24 ± 0.11	0.063
	Y-axis	9.27 ± 1.77	9.29 ± 1.50	0.726	9.09 ± 1.75	0.489
	Z-axis	2.40 ± 0.37	2.23 ± 0.41	0.631	2.10 ± 0.46	0.564
Knee joint	X-axis	0.22 ± 0.07	0.09 ± 0.08	0.161	0.05 ± 0.06	0.063
	Y-axis	0.29 ± 0.13	0.40 ± 0.22	0.554	0.36 ± 0.06	0.700
	Z-axis	−0.83 ± 0.40	−0.85 ± 0.48	0.928	−0.79 ± 0.36	0.846
Ankle joint	X-axis	0.04 ± 0.02	0.23 ± 0.18	0.251	−0.10 ± 0.20	0.076
	Y-axis	0.33 ± 0.28	0.57 ± 0.57	0.372	0.47 ± 0.31	0.091
	Z-axis	−0.27 ± 0.04	−0.42 ± 0.39	0.561	−0.40 ± 0.31	0.657

Note: X-axis is the sagittal axis, Y-axis is the vertical axis, and Z-axis is the frontal axis.

#### 3.1.2 Comparison of joint moment of the lower limbs in three environments

As shown in Table 3, the joint moments of the wheelchair groups were compared and analyzed in the three environments: flat ground, slope, and stairs. When using a wheelchair, no significant differences were observed in hip abduction/adduction (abd/add), hip flex/ext, knee flex/ext, or ankle flex/ext moments in slope and stair environments compared to flat ground environments.

**Table 3:** Mean and standard deviation (mean ± SD) of the maximum joint moment of the hip, knee, and ankle in three environments. Units: Nm/BW.

Joint moment	Group	Flat ground	Slope	p-value	Stairs	p-value
Hip abd/add	Wheelchair	0.09 ± 0.04	0.08 ± 0.06	0.728	0.09 ± 0.04	0.860
Hip flex/ext	Wheelchair	0.18 ± 0.13	0.25 ± 0.07	0.534	0.29 ± 0.06	0.606
Knee flex/ext	Wheelchair	0.03 ± 0.01	0.05 ± 0.03	0.231	0.04 ± 0.03	0.361
Ankle flex/ext	Wheelchair	0.06 ± 0.03	0.03 ± 0.02	0.085	0.03 ± 0.01	0.054

### 3.2 Comparison of lower limb muscle strength in three environments

As shown in Table 4 and Fig 5, we compared the lower limb muscle strength of the wheelchair group in three different environments. As for the wheelchair group, there were no significant differences in muscle strength in slope and stair environments compared to flat ground environments.

**Table 4:** Mean and standard deviation (mean ± SD) of the maximum of lower limb muscle strength in three environments. Units: N/BW.

Muscle	Flat ground	Slope	p-value	Stair	p-value
Adductor longus	34.95 ± 29.72	34.50 ± 7.57	0.640	28.19 ± 10.00	0.447
Sartorius	73.22 ± 33.15	74.67 ± 39.42	0.759	64.49 ± 26.31	0.640
Gluteus maximus	2.56 ± 0.14	2.89 ± 0.89	0.773	3.56 ± 1.21	0.477
Gluteus minimus	15.08 ± 5.79	17.97 ± 8.80	0.313	19.65 ± 7.75	0.279
Popliteal muscle	0.19 ± 0.34	0.10 ± 0.19	0.164	0.18 ± 0.44	0.329
Soleus	0.09 ± 0.12	0.39 ± 0.01	0.062	0.16 ± 0.04	0.103
Rectus femoris	70.64 ± 22.5	80.57 ± 21.16	0.298	81.54 ± 22.26	0.159
Biceps femoris	14.11 ± 7.58	11.95 ± 9.24	0.641	11.72 ± 5.72	0.660
Vastus lateralis	11.84 ±2.07	12.80 ± 1.49	0.876	14.46 ± 2.74	0.675
Vastus medialis	1.10 ± 0.90	0.65 ± 0.27	0.338	1.04 ± 0.52	0.829
Tibialis anterior	5.60 ± 5.36	4.38 ± 4.05	0.649	5.01 ± 4.75	0.770
Gastrocnemius	0.42 ± 0.40	0.96 ± 1.20	0.324	1.84 ± 1.10	0.093

### 3.3 Finite element analysis of skeletons in three environments while using wheelchairs

In the established finite element model, the maximum joint force of the participant during the entire movement process is taken as the load for static simulation calculation. The shear force from AnyBody’s inverse dynamics calculation decides the load direction. Load facets refer to the articular facets in contact between joints during human activities.

#### 3.3.1 Finite element stress analysis of the skeleton

**Fig 4:**
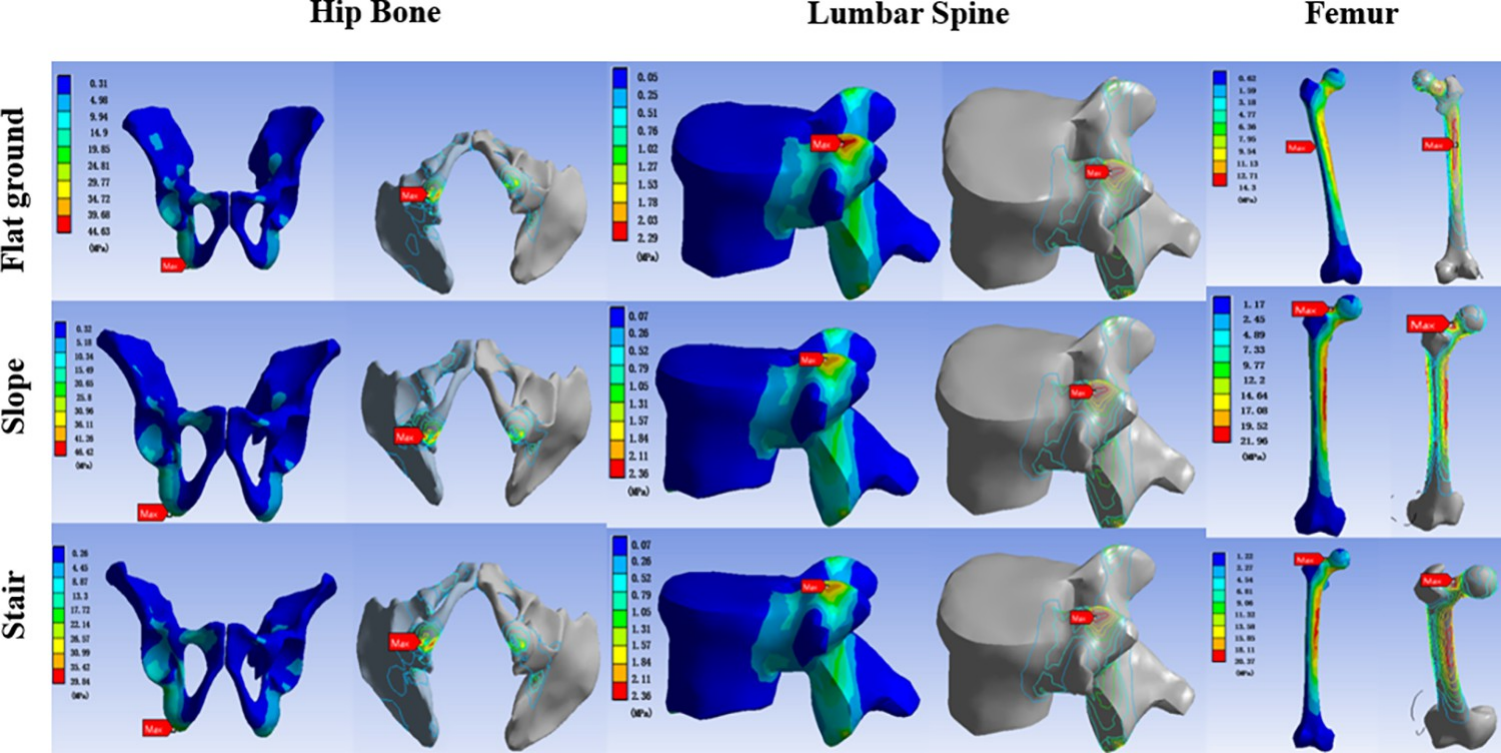
Equivalent stress distribution of the hip bone, lumbar spine, and femur in the stair-climbing wheelchair. The warmer the color, the greater the stress. The red arrow indicates the maximum stress.

**Fig 5:**
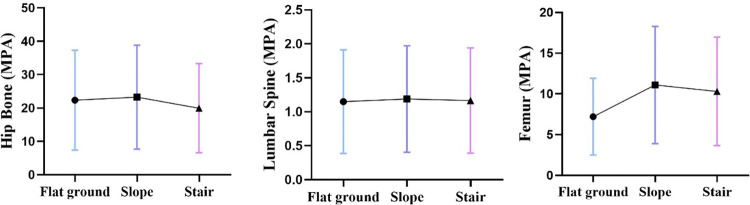
Change in mean values of maximum equivalent stress of hip, lumbar, and femur stress in three environments (flat ground in blue; slope in purple; and stairs in pink).

We conducted finite element analysis on the participants using the wheelchair in the three environments, and the results are shown in Fig 4. Fig 5 presents the minimum-maximum distribution of skeletal stress. The maximum stress of the hip bone was located at the ischial tuberculum under all three environments. The stress in the slope environment was slightly higher than that in the flat ground environment, and the stress in the stairs environment was slightly lower. The stress position of the fifth lumbar vertebra was at the end of the facet of the lumbar joint, the average stress values of the three environments were 1.14 MPa, 1.18 MPa, and 1.16 MPa, and the distribution of primary stress did not change. The maximum stress of the femur in the flat ground environment was located at the back and upper part of the femur body, whereas the maximum stress of the femur was located at the neck of the femur in both the slope and stairs environments, and the stress values in these environments were significantly higher than in the flat ground environment. According to the overall stress change curves in Figs 4 and 5, among the three environments of flat ground, slope, and stairs, the stress values of the three bones exhibited no significant changes, and the stress distributions were relatively stable.

#### 3.3.2 Finite element deformation displacement analysis of the skeleton

**Fig 6:**
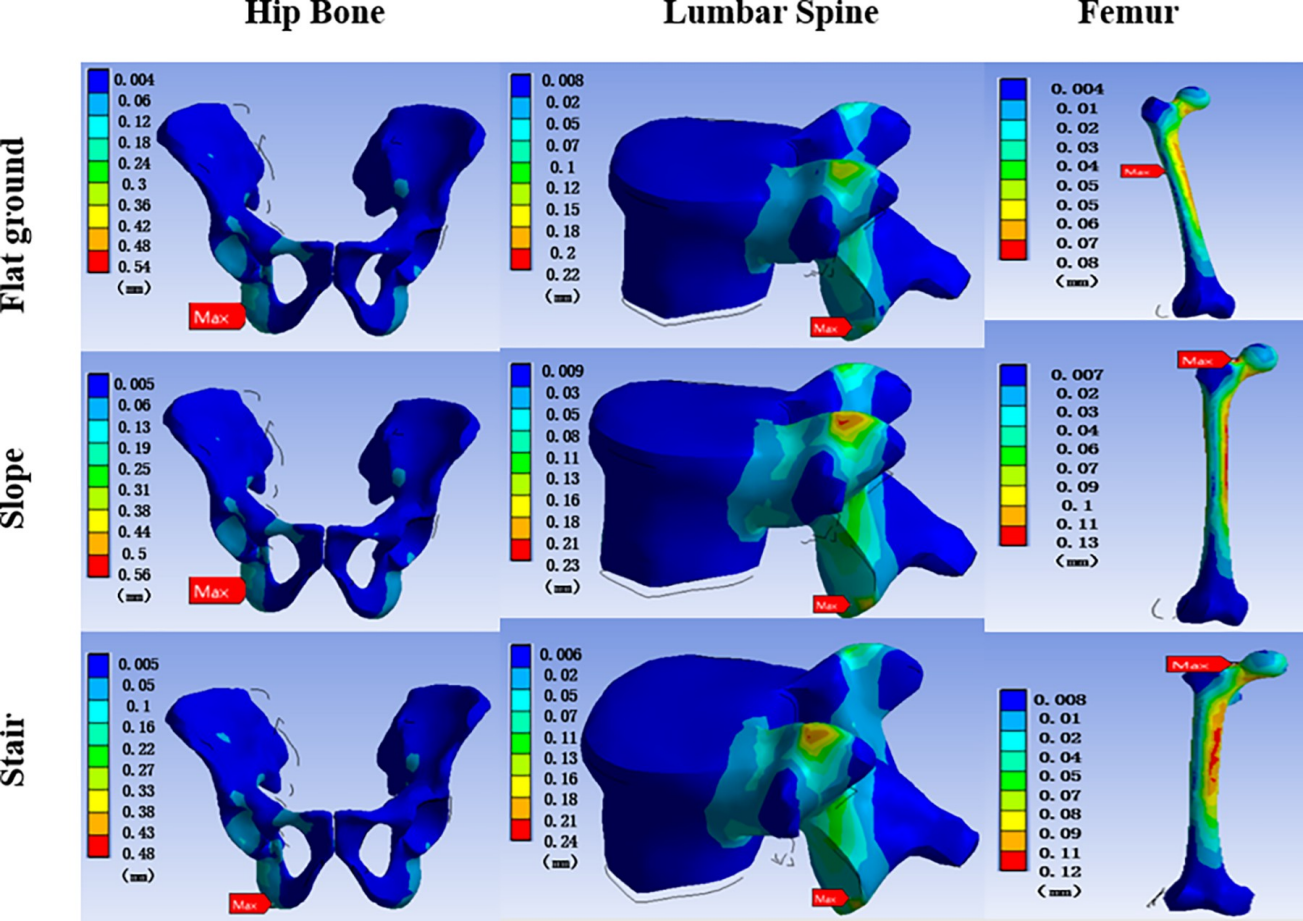
Resultant displacement distributions of the hip bone, lumbar spine, and femur in the stair-climbing wheelchair. The red arrow indicates the maximum displacement position.

**Fig 7:**
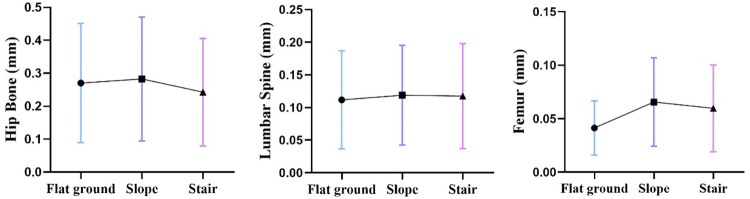
Changes in mean values of maximum resultant displacement in stair-climbing wheelchair in three environments (flat ground in blue; slope in purple; and stairs in pink).

We compared the bone stress of the wheelchair group among the three environments. As shown in Figs 6 and 7, the results of the skeletal strain analysis show that when using a stair-climbing wheelchair, the hip, fifth lumbar vertebra, and femur displacement were not significantly different in the different environments.

The maximum displacement and maximum stress of the hip and femur were consistent. The maximum displacement of the fifth lumbar spine was located at the lower end of the lumbar joint. Comparing the displacement of the bones, the displacement of the hip was the largest, followed by the displacement of the lumbar vertebra and the displacement of the femur. The overall displacement distributions of the three bones were relatively stable.

## 4. Discussion

In daily life, the energy consumption of walking is high, and walking can be difficult or dangerous for older adults [27]. Wheelchairs are essential mobility devices for many people, and they can effectively reduce the muscle power required for mobility [28]. Metabolic cost reduction is optimized through wheelchair-related equipment. In this study, the daily use of a new electric stair-climbing wheelchair was investigated in detail in terms of its application in three environments (flat ground, slope, and stairs) encountered in daily life.

In this paper, we found that there were no significant differences in lower limb joint stresses and muscle strength when using wheelchairs in the three environments. Queen [29] discovered that the muscle strength and stability of the lower limbs decrease significantly with age, resulting in the increased risk of falling while ascending stairs due to poor muscle stability and electric stair-climbing wheelchairs can reduce the muscle strength required to climb stairs. That conclusion was verified in this study. For example, whereas lower limb Joint force, Joint moment and muscle strength did not change among the three environments when using the stair-climbing wheelchair. Therefore, the stair-climbing wheelchair studied in this paper is proven practical in different environments.

In addition, based on the finite element analysis of the stress and strain of a healthy woman’s fifth lumbar vertebra, hip, and femur, we analyzed the bone stress involved in sitting in the stair-climbing wheelchair in the three environments. The maximum stress of the fifth lumbar vertebra was on the upper end of the lumbar articular facet in all three environments. The maximum displacement was on the lower end, which is the same result as that in the study by Guo [30], because the waist is in the vertical posture during the normal sitting posture, and the lumbar posture in the vertical posture will increase the stress in the anterior and lateral discs of the lumbar vertebra. This study found that the maximum stress reached 7.21 MPa, and the maximum stress should not be exceeded to maintain the comfort of the sitting position. For example, Ciaccia & Sznelwar [31] and Conine [32] stated that the skeletal stress threshold, where the skeleton should not exceed its maximum stress threshold in motion, would otherwise block the capillaries, depriving tissues of oxygen, and the body would then experience discomfort. In this study, the maximum stresses of the fifth lumbar spine were 2.29 MPa (flat ground), 2.36 MPa (slope), and 2.32 MPa (stairs), all of which were lower than the maximum stress of 7.21 MPa, and the distribution of lumbar pressure was consistent with that found by Berkson [33]. Based on our analysis of the pressure on the hips, we conclude that when participants used the wheelchair, the most significant stress was concentrated on the ischial tubercle in all three environments, as the ischium is in the top support position when sitting. Regarding femoral stress, we found that the maximum stress of the femur was in the upper part of the femur; in the case of the slope and stairs, the maximum stress was in the femoral neck, and the stress was mainly produced by the reaction moment and the muscle moment. This is because the pressure distribution of the wheelchair is mainly on the muscles around the hips and the hips, and during ascent, the legs are suspended and cannot be appropriately supported, creating stress in the neck of the femur.

Finally, according to the three skeletal stress and strain displacement diagrams, the stress distributions of the fifth lumbar vertebra, hip bone, and femur stayed approximately the same under the different environments. The strain displacement was not significantly changed, and the strain displacement or stress did not increase with the continuous increase in the steepness of the slope or stairs, which is a good indication that the stair-climbing wheelchair user’s body will remain stable while ascending slopes and stairs. This wheelchair can adjust the body posture according to environmental changes and offers good stability and comfort. In addition, previous studies have mainly focused on the characteristics of kinematics. This paper verifies the consistency of the data by combining dynamics and finite element simulation parameters, allowing people to use climbing wheelchairs for adequate and reasonable scientific verification.

## 5. Conclusion

As the most efficient mobility device for older adults and the physically disabled, wheelchairs can play a vital auxiliary role in daily life. This paper investigates and verifies the dynamics of the lower extremities while using a stair-climbing wheelchair in three environments encountered in daily life. Comparative analysis shows that with an independently designed wheelchair in this experiment, the bodies of the wheelchair users move more smoothly and stably in all three environments and show no changes among the three environments. However, there is no further comparative study on different types of stair-climbing wheelchairs. Future work will verify the physical force and stability of different electric wheelchairs by comparing different climbing wheelchairs, guide the improvement of the stability and comfort of wheelchairs, and propose design suggestions from the perspective of human force.
